# Anticancer Activity of Bacterial Proteins and Peptides

**DOI:** 10.3390/pharmaceutics10020054

**Published:** 2018-04-30

**Authors:** Tomasz M. Karpiński, Artur Adamczak

**Affiliations:** 1 Department of Genetics and Pharmaceutical Microbiology, Poznań University of Medical Sciences, Święcickiego 4, 60-781 Poznań, Poland; 2 Department of Botany, Breeding and Agricultural Technology of Medicinal Plants, Institute of Natural Fibres and Medicinal Plants, Kolejowa 2, 62-064 Plewiska, Poland; artur.adamczak@iwnirz.pl

**Keywords:** anticancer, bacteria, proteins, anticancer antibiotics, anticancer enzymes, nonribosomal peptides, bacteriocins, bacterial toxins

## Abstract

Despite much progress in the diagnosis and treatment of cancer, tumour diseases constitute one of the main reasons of deaths worldwide. The side effects of chemotherapy and drug resistance of some cancer types belong to the significant current therapeutic problems. Hence, searching for new anticancer substances and medicines are very important. Among them, bacterial proteins and peptides are a promising group of bioactive compounds and potential anticancer drugs. Some of them, including anticancer antibiotics (actinomycin D, bleomycin, doxorubicin, mitomycin C) and diphtheria toxin, are already used in the cancer treatment, while other substances are in clinical trials (e.g., p28, arginine deiminase ADI) or tested in in vitro research. This review shows the current literature data regarding the anticancer activity of proteins and peptides originated from bacteria: antibiotics, bacteriocins, enzymes, nonribosomal peptides (NRPs), toxins and others such as azurin, p28, Entap and Pep27anal2. The special attention was paid to the still poorly understood active substances obtained from the marine sediment bacteria. In total, 37 chemical compounds or groups of compounds with antitumor properties have been described in the present article.

## 1. Introduction

Cancer belongs to the main reasons of morbidity and mortality in the world. In the year 2012, approximately 14 million new cancer cases were detected [1]. In 2015, cancer was responsible for 8.8 million deaths. Lung, liver, colorectal, stomach and breast cancers were the most common causes of death [2,3]. To reduce premature mortality from cancer, the resolution: ‘Cancer Prevention and Control in the Context of an Integrated Approach’ (WHA70.12) was passed in 2017 by the World Health Assembly [4].

Cancerous cells are altered host cells without the natural mechanisms controlling their normal growth. Oncogenesis can be caused by environmentally induced or inherited genetic mutations. It leads to inhibition of cell reaction to the control mechanisms of normal growth and gives rise to the rapid development of cell clones producing neoplasm [5]. Treatment of cancer involves apoptosis induction and tumour-cell proliferation inhibition [6].

According to Hanahan and Weinberg [7], cancer cells exhibit six important changes in their own physiology: (1) self-sufficiency in signals of growth, (2) insensitivity to signals inhibiting growth, (3) resistance to apoptosis, (4) unlimited proliferative potential, (5) sustained angiogenesis and (6) metastasis. One of the available treatments for cancer is chemotherapy, which very often belongs to the main choice of treatment. Unfortunately, chemotherapy can lead to damage of healthy cells and tissues or development of drug resistance [8].

**Figure 1:**
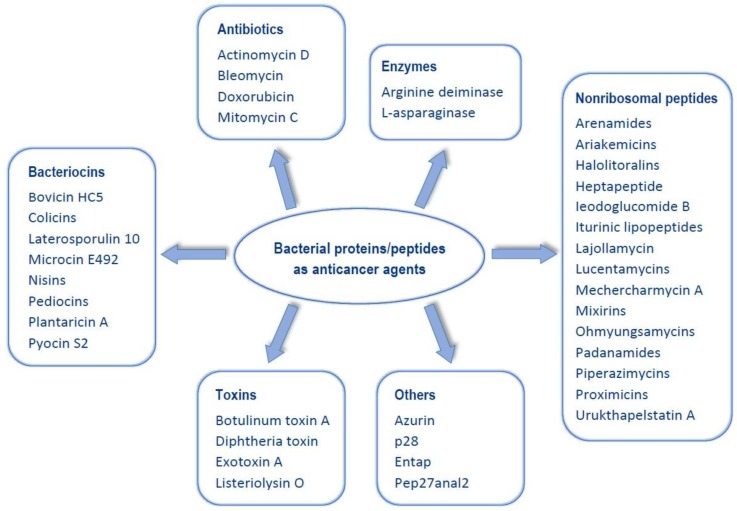
Division of the described anticancer proteins and peptides.

The most known examples of usage of bacteria and their metabolites for the cancer treatment are investigations made by William Coley [9], who utilized *Streptococcus pyogenes* and *Serratia marcescens* supernatants in the treatment of patients with unresectable tumours. This mixture, called today as ‘Coley’s toxins’, was used in approximately 1200 patients with malignancy. Cancer regression in 52 cases, including complete cure of 30 patients, was observed. Mechanism of this reaction has now been partially recognized. Microbial infections can activate macrophages and lymphocytes and induce the cytotoxic substance production, particularly tumour necrosis factor α (TNF-α) [10]. Currently, bacterial proteins and peptides are important as antiproliferative agents. Some of these are already used in cancer treatment, others are in human clinical trials or studied in vitro. In this paper, main anticancer proteins and peptides of bacterial origin are presented. Suggested division of the described proteins and peptides is shown in Figure 1.

## 2. Antibiotics

**Figure 2:**
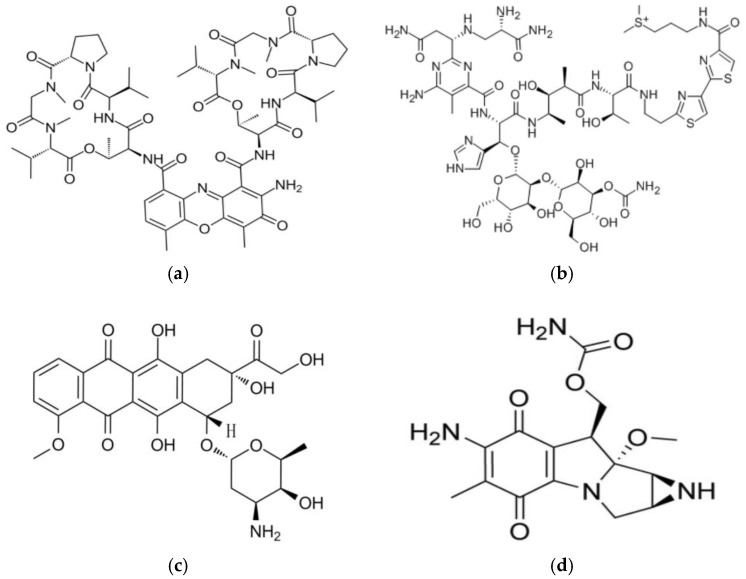
Chemical structures of anticancer antibiotics: (**a**) Actinomycin D; (**b**) Bleomycin A2; (**c**) Doxorubicin; (**d**) Mitomycin C.

According to *Encyclopaedia Britannica* [11], antibiotics are the chemical compounds produced mostly by the microorganisms and injurious to other organisms from this group. It has been observed that some of the antibiotics also have anticancer activity and recently they have been used mainly as antitumor drugs. The origin and biological target of four antibiotics already utilized in medicine as chemotherapeutic drugs are presented in Table 1 and their chemical structures in Figure 2.

### 2.1. Actinomycin D

Actinomycin D (dactinomycin) is a well-known antibiotic produced by *Actinomyces antibioticus* that exhibits antibacterial and antitumor activity. This drug has a chemical formula of C_62_H_86_N_12_O_16_ and a molecular weight of 1.26 kDa [15]. Actinomycin D has several mechanisms of its cytotoxic and antitumor action: intercalation to DNA and the stabilization of cleavable complexes of topoisomerases I and II with DNA, photodynamic activity and free radical formation [27]. Presented drug blocks both DNA and RNA expression and as a consequence protein synthesis. Therefore, it induces cellular p53-independent apoptosis [28]. Actinomycin D is effective in the treatment of Wilms cancer, Ewing sarcoma, neuroblastomas and trophoblastic tumours, primarily in children. It is also used as a tool in the study of many cellular processes, such as the biosynthesis of cell macromolecules, RNA transport or viral replication [15,29]. Following drugs containing actinomycin D: Actinomycin D, Cosmegen and Lyovac are available, among others, on the market [30].

### 2.2. Bleomycin

Bleomycin (BLM) is a mixture of glycopeptide antibiotics with cytotoxic properties, obtained from *Streptomyces verticillus*. Bleomycin A2 has a chemical formula of C_55_H_84_N_17_O_21_S_3_ and a molecular mass of 1.42 kDa, while in the case of bleomycin B2 it is C_55_H_84_N_20_O_21_S_2_ and 1.43 kDa [31]. Bleomycin induces oxygen- and metal ion-dependent cleaving of DNA. BLM binds DNA and Fe(II) and hydroxyl radicals are released under the influence of molecular oxygen, causing as a consequence DNA damage and Fe(II) oxidation. BLM is used in the treatment of head and neck squamous cell carcinomas, Hodgkin’s disease, non-Hodgkin’s lymphoma, testicular carcinomas, ovarian cancer and malignant pleural effusion [16,17]. Drugs containing bleomycin: Bleomycin USP and Blenoxane are available [32,33].

### 2.3. Doxorubicin

Doxorubicin (DOX) is an anthracycline antibiotic with antitumor activity, originally isolated from *Streptomyces peucetius* var. *caesius*. It is an amphiphilic molecule containing two parts: water-insoluble aglycone (adriamycinone: C_21_H_18_O_9_) and water-soluble, amino-sugar functional group (daunosamine: C_6_H_13_NO_3_) [20]. DOX acts on the nucleic acids of dividing cells by two main mechanisms: (i) intercalation between the base pairs of the DNA strands and inhibition of the synthesis of DNA and RNA in rapidly growing cells by blocking the replication and transcription processes [34]; and (ii) generation of iron-mediated free radicals, causing oxidative damage to cellular membranes, proteins and DNA [35]. DOX belongs to the most commonly used drugs in chemotherapy. Nowadays, this substance is recommended by the Food and Drug Administration (FDA) in the case of acute lymphoblastic leukaemia, acute myeloblastic leukaemia, Wilms’ tumour, neuroblastoma, soft tissue and bone sarcomas, breast carcinoma, ovarian carcinoma, transitional cell bladder carcinoma, thyroid carcinoma, gastric carcinoma, Hodgkin’s disease, malignant lymphoma and bronchogenic carcinoma in which the small-cell histologic type is the most responsive compared with other cell types [23]. Preet et al. [36] demonstrated that combining doxorubicin with nisin may improve the treatment efficiency of the skin cancers. Adriblastine PFS, Caelyx, Doxorubicin medac, Doxorubicin-Ebewe, Doxorubicinum Accord and Myocet belong to the drugs containing doxorubicin [37].

### 2.4. Mitomycin C

Mitomycin C was isolated from a strain of actinomyces, *Streptomyces caespitosus*. Its molecular formula is C_15_H_18_N_4_O_5_ and a molecular weight of 334 Da [24]. This antitumor agent inhibits DNA synthesis by binding to DNA on the path of alkylation, which results in crosslinking of strands of double helical DNA [38]. Mitomycin C is utilized in the treatment of cancers of the head and neck, lungs, breast, cervix, bladder, colorectal and anal, hepatic cell carcinoma and melanoma in addition to the stomach and pancreatic cancer [25]. Among others, Mitomycin Accord and Mitomycin C Kyowa are available on the market [39].

## 3. Bacteriocins

**Figure 3:**
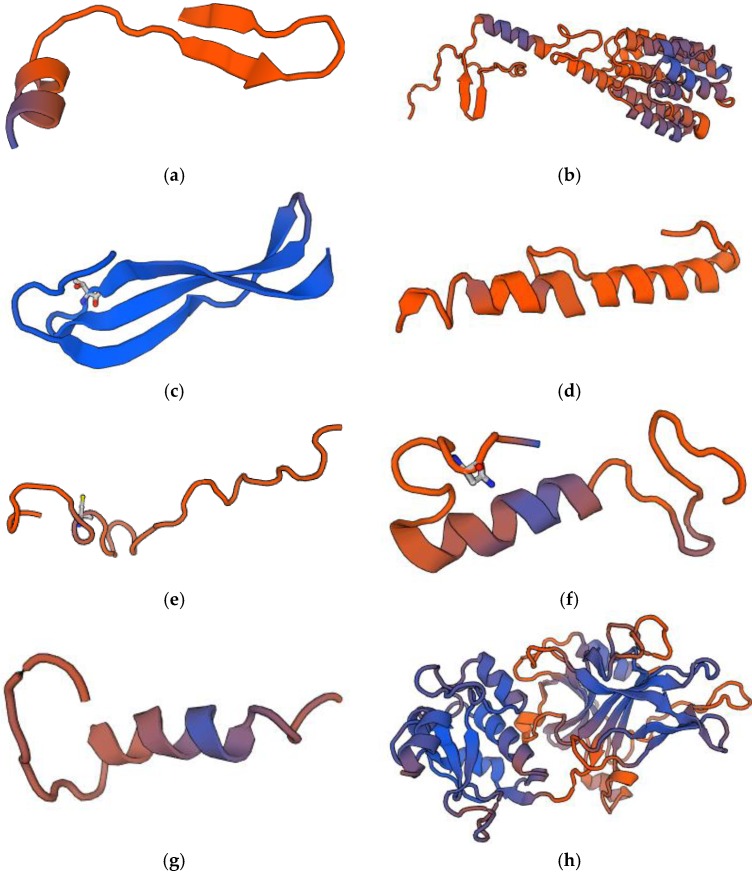
Structure models of anticancer bacteriocins: (**a**) Bovicin HC5; (**b**) Colicin E1; (**c**) Laterosporulin 10; (**d**) Microcin E492; (**e**) Nisin A; (**f**) Pediocin CP2; (**g**) Plantaricin A; (**h**) Pyocin S2 (orig.). Bacteriocin sequences were downloaded from BACTIBASE [61] and UNIPROT [62] and modelling server SWISS-MODEL [63,64] was used to the visualization of the bacteriocin structures.

Bacteriocins constitute a heterogeneous group of ribosomally synthesized bacterial peptides or proteins with antimicrobial properties [40]. Some of them also show anticancer activity [41,42,43]. There are four classes of bacteriocins secreted by Gram-positive bacteria. Group I includes antibiotics or thermostable peptides with a molecular mass below 10 kDa. They come under posttranslational modification and comprise unusual amino acids, including lanthionine (Lan), methyllanthionine (MeLan), dehydroalanine (Dha), dehydrobutyrine (Dhb) and D-alanine (D-Ala). Class II contains thermostable bacteriocins without lanthionine. The molecular weight of these bacteriocins is below 10 kDa. Pediocin-like bacteriocins, dipeptide bacteriocins and cyclic peptides belong to this class. In turn, group III includes thermolabile bacteriocins with a molecular mass above 10 kDa. These substances are subdivided into bacteriolysins and nonlytic proteins. Class IV consists of bacteriocins requiring the presence of lipid or carbohydrate moieties for full activity [40,44,45]. Bacteriocins isolated from Gram-negative bacteria are microcins secreted by Enterobacteriaceae with a molecular weight below 10 kDa and plasmid-encoded colicins with a molecular weight above 20 kDa [40,46]. The origin and biological activity of anticancer bacteriocins are shown in Table 2. Structure models of some anticancer bacteriocins are presented in Figure 3.

### 3.1. Bovicin HC5

The lantibiotic bovicin HC5 is secreted by *Streptococcus bovis* and has a molecular mass of 2.4 kDa. This compound indicates structural and functional similarities to the nisin [41]. Paiva et al. [47] showed in vitro the bovicin HC5 cytotoxicity against human breast adenocarcinoma (MCF-7) and human liver hepatocellular carcinoma (HepG2) with a half maximal inhibitory concentration (IC_50_) of 279.4 and 289.3 µM, respectively. At the maximum tested dose of bovicin (350 µM), the cell line viability was less than 20% [47].

### 3.2. Colicins

Colicins A, E1 and E3 are produced by *Escherichia coli* and have molecular sizes: more than 20, 57 and 9.8 kDa, respectively [41]. Colicins E1 and E3 exhibited cytotoxic activity against BM2 cells (chicken monoblasts transformed with the v-myb oncogene of avian myeloblastosis virus). The maximum effect was reached when the cells were exposed to colicin E1 (1.25 µg/mL) for 48 h. Authors demonstrated that colicin E3 did not result in modifications of cell cycle. It suggests that the above-mentioned substance kills cells on the path of necrosis rather than apoptosis [65]. Chumchalova and Smarda [48] investigated four colicins (A, E1, U, E3) in terms of their inhibition activity against 11 cancer cell lines. Colicin E1 and A inhibited 10 cell lines: breast carcinoma (MCF7, ZR75, BT549, BT474, MDA-MB-231, SKBR3 and T47D), osteosarcoma (HOS), leiomyosarcoma (SKUT-1) and fibrosarcoma (HS913T). Only the colon carcinoma line (HT29) was insensitive to colicin E1. Colicin E1 showed 50% inhibition of fibrosarcoma (HS913T) and 17–40% inhibition of other cancer cell lines. Colicin A indicated from 16 to 56% inhibition of cancer cell lines and 36% inhibition of normal diploid fibroblasts with wild-type p53 (MRC5). Colicin E3 demonstrated no significant inhibition activity against tested cancer cells [48].

### 3.3. Laterosporulin 10

Laterosporulin 10 (LS10) is a defensin-like peptide of *Brevibacillus* sp. inhibiting microbial pathogens. The anticancer activity of this substance was investigated using normal prostate epithelium cell line (RWPE-1) and five different human cancer cell lines including cervical cancer (HeLa), embryonic kidney cancer (HEK293T), fibrosarcoma (HT1080), lung carcinoma (H1299) and breast cancer (MCF-7). Authors observed a dose-dependent cytotoxic action on all tumour cell lines with maximum activity at 10 μM and the highest activity against MCF-7 cells. Simultaneously, LS10 did not have any cytotoxic properties against normal cells up to 15 μM, whereas significant cytotoxicity was detected against cancer cells at this concentration. At lower doses, this substance caused apoptosis of cancerous cells, while at higher doses it resulted in necrotic death of them [49].

### 3.4. Microcin E492

Microcin E492 (M-E492) is a bacteriocin produced by *Klebsiella pneumoniae* RYC492 and it has a molecular mass of 7.9 kDa. The cytotoxicity of M-E492 was detected in the case of various malignant human cell lines, including cervical adenocarcinoma (HeLa), acute T cell leukaemia (Jurkat), B cell line originated from Burkitt’s lymphoma (Ramos) and B-lymphoblastoid cell lines transformed by infection with Epstein-Barr virus (RJ2.25, a variant of the Raji B-LCL). At the same time, no effect was determined against human endothelial cells from human tonsils (AMG-3) and a monocyte-macrophage cell line (KG-1) [50]. Jurkat cell line was the most sensitive to microcin E492, with 96% viability decrease after 24 h of incubation. At a low concentration (5–10 μg/mL), M-E492 induced apoptosis of cancer cells, while at a higher concentration (20 μg/mL) it caused necrosis of them. It was reported that M-E492 leaded to the morphological and biochemical modifications during apoptosis such as: cell shrinkage, fragmentation of DNA, extracellular exposure of phosphatidylserine, caspase activation, decline of potential of mitochondrial membrane and also release of calcium ions from intracellular stores [50].

### 3.5. Nisins

Nisin is a 34-amino acid polycyclic antibacterial peptide of *Lactococcus lactis*. Nisin has a broad-spectrum antibacterial effect and inhibits both Gram-positive and Gram-negative bacteria. Additionally, this substance is safe for human consumption, therefore it has been approved for use as a food preservative for over 50 years. Nisin (E 234) is authorized for food preservation in the USA by FDA and in the European Union by Directive 95/2/EC [40]. Joo et al. [53] presented the anticancer activity of this substance and found that nisin A inhibits tumorigenesis of head and neck squamous cell carcinoma (HNSCC). Treatment of three different HNSCC cell lines (UM-SCC-17B, UM-SCC-14A and HSC-3) with increasing concentrations of nisin (from 5 to 80 µg/mL) induced growing level of DNA fragmentation or apoptosis after 24 h of treatment. On the other hand, primary oral keratinocytes did not show higher DNA fragmentation. Nisin has an impact on induction of apoptosis, stopping of cell cycle and reduction of HNSCC cell proliferation, in part, through cation transport regulator homolog 1 (CHAC1), a proapoptotic cation transport regulator and through a concomitant CHAC1-independent influx of extracellular calcium. Nisin also limited HNSCC tumorigenesis in a mouse model [39]. Paiva et al. [47] observed that for human cell lines of breast adenocarcinoma (MCF-7) and liver hepatocellular carcinoma (HepG2) treated with nisin, the obtained IC_50_ value was 105.46 and 112.25 µM, respectively. Also, nisin ZP significantly increased apoptosis of HNSCC cells (UM-SCC-17B and HSC-3). Nisin ZP was suggested by authors for the treatment of HNSCC, through the promotion of apoptosis of HNSCC cells and suppression of their proliferation as well as inhibition of angiogenesis, orasphere formation and tumorigenesis in vivo [54].

### 3.6. Pediocins

Pediocins belong to the class IIa of bacteriocins. Pediocin CP2 is produced by *Pediococcus acidilactici* MTCC 5101 and it is built from 44 amino acids [55]. Kumar et al. [56] studied cytotoxic effect of native pediocin and recombinant rec-pediocin on several cancerous cell lines. A mouse spleen lymphoblast cell line (Sp2/O-Ag14) exhibited the highest sensitivity to rec-pediocin CP2, while cell lines of mammary gland adenocarcinoma (MCF-7), hepatocarcinoma (HepG2) and cervical adenocarcinoma (HeLa) were sensitive at different degree to the action of native and rec-pediocin. After 48 h of rec-pediocin treatment, epithelial tissue models had only a low level of viability. Total cell viability of Sp2/O-Ag14 decreased to 0% due to acute toxicity of 25 µg/mL of rec-pediocin, whereas cell lines with native pediocin retained 26.7% viability. The viability of cell lines treated with 25 µg/mL of rec-pediocin and native pediocin CP2 was 2.1 and 10.7% for MCF-7 as well as 5.5 and 1.2% for HepG2 cells, respectively. HeLa cells showed lower sensitivity towards rec-pediocin with comparing to other tumour cell lines [56]. In studies of undialyzed (1600 AU/mL) and dialyzed (800 AU/mL) fractions of bacteriocin from *P. acidilactici* K2a2-3, authors observed growth inhibition of 55 and 53.7% of human colon adenocarcinoma cells (HT29), respectively. In turn, undialyzed bacteriocin fraction inhibited the growth of 52.3% of human cervical carcinoma cells (HeLa) and only 15.6% were inhibited by dialyzed fraction [57].

### 3.7. Plantaricin A

Plantaricin A is a bacteriocin of *Lactobacillus plantarum* C11 and its molecular weight reaches 2.4 kDa. In the case of artificially synthesized plantaricin A, the cytotoxicity against the human T cell leukaemia (Jurkat) was determined in vitro. It was shown that bacteriocin dose of 25 µM at 20 °C caused 75% loss in the cell viability, while at 37 °C it decreased by 55%. Plantaricin A induced apoptosis and necrosis of Jurkat cell line which were observed as a fragmentation of cell nuclei and plasma membrane. Tested substance had also impact on increasing of intracellular concentration of caspase-3 in cancerous cells [58].

### 3.8. Pyocins

Pyocins are secreted by more than 90% of *Pseudomonas aeruginosa* strains. Additionally, each strain can produce several different compounds from this group [66]. Investigations of Abdi-Ali et al. [59] showed the cytotoxicity of partially purified pyocin and pyocin S2 obtained from *P. aeruginosa* 42A on human hepatocellular carcinoma (HepG2) and human immunoglobulin-secreting cell line derived from multiple myeloma (Im9). Both pyocins were totally non-toxic to normal human foetal foreskin fibroblast cell line (HFFF). Im9 indicated greater sensitivity than HepG2 and the highest inhibition of growth (80%) was determined at a maximum concentration of pyocin (50 U/mL) after 5 days of cell incubation [59]. In turn, Watanabe and Saito [46] presented the cytotoxic action of pyocin S2 on cell lines of cervical adenocarcinoma (HeLa) and embryonal carcinoma of ovary (AS-II) as well as simian virus 40-transformed mouse kidney cells (mKS-A TU-7) and normal mice cells (BALB/3T3). On the other hand, there was no cytotoxic action on cells of metastatic lymph node of gastric cancer (HCG-27) and also normal cells of rat kidney and human lung [60].

## 4. Enzymes

**Figure 4:**
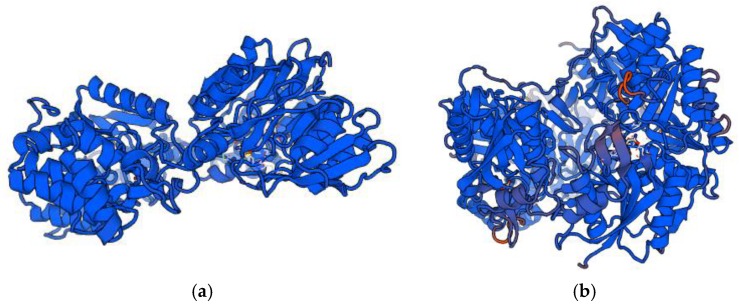
Structure models of anticancer antibiotics: (**a**) Arginine deiminase; (**b**) l-asparaginase (orig.). Sequences were downloaded from UNIPROT [62] and modelling server SWISS-MODEL [63,64] was used to visualization of the antibiotic structures.

Some of the bacterial enzymes, like arginine deiminase and l-asparaginase, are utilized in the treatment of selected cancer diseases. The source and biological target of antitumor bacterial enzymes are presented in Table 3 and their structure models are shown in Figure 4.

### 4.1. Arginine Deiminase

Arginine deiminase (ADI) is an enzyme secreted by *Mycoplasma hominis* or *M. arginini* that degrades arginine to citrulline in vivo, releasing ammonia [67]. Recent studies are based on pegylated arginine deiminase (ADI-PEG20). The efficacy of ADI-PEG20 is directly correlated with the deficiency of argininosuccinate synthetase (ASS) [69]. Arginine deiminase in its native form is strongly antigenic with a half-life of 5 h [81]. ADI-PEG20 (arginine deiminase conjugated to 20,000 mw polyethylene glycol) decreases antigenicity and increases serum half-life [82]. Arginine deiminase may control the growth of argininosuccinate synthase deficient or arginine auxotrophic hepatocellular carcinoma (HCC). The pegylated ADI shows moderate disease-stabilizing activity in HCC and constitutes a promising drug utilizing a high enzymatic deficiency in HCC. This is a safe and well-tolerated therapy, which may benefit patients with unresectable hepatocellular carcinoma. Recently, usage of arginine deiminase as a drug is in the phase II clinical study [68]. Also, prostate cancer cells (CWR22Rv1) are susceptible to ADI-PEG20 in vitro. Apoptosis, observed after 96 h of treatment by 0.3 mg/mL ADI-PEG20 is caspase-independent. The effect of ADIPEG20 in vivo reveals reduced tumour activity and growth. Additionally, authors describe autophagy induced by single amino acid depletion by ADI-PEG20. Autophagy was reported within 1 to 4 h of 0.3 mg/mL ADI-PEG20 treatment and it was an initial protective response to ADI-PEG20 in CWR22Rv1 cells [69]. A significant reaction, with cytotoxicity up to 50%, was also detected in the case of 4 glioblastoma cell lines (HROG02, HROG05, HROG10 and HROG17). The anticancer effect of ADI was independent of apoptosis, while reduction of cell proliferation was observed [70].

### 4.2. l-asparaginase

l-asparaginase (ASNase) enzyme was obtained from *Escherichia coli* or *Erwinia* species. The anti-tumour action of bacterial ASNases is caused by their ability to reduce asparagine blood concentration causing a selective inhibition of growth of sensitive malignant cells [73]. Panosyan et al. [74] presented that ASNase treatment in vitro resulted in dose-dependent growth inhibition of the following brain tumour cell lines: a paediatric medulloblastoma (DAOY), p53 and PTEN null human glioblastomas (GBM-ES and U87). Recently, ASNase has been utilized in the treatment of acute lymphoblastic leukaemia (ALL), myeloblastic leukaemia, Hodgkin and non-Hodgkin lymphomas, myelosarcoma, multiple myeloma, extranodal NK/T cell lymphoma and ovarian carcinomas [75,76,77]. *Erwinia* asparaginase should be used for the second- or third-line treatment of acute lymphoblastic leukaemia (ALL), depending upon regulatory requirements, in patients developing hypersensitivity to *E. coli* asparaginase preparations [83].

## 5. Nonribosomal Peptides (NRPs)

**Figure 5:**
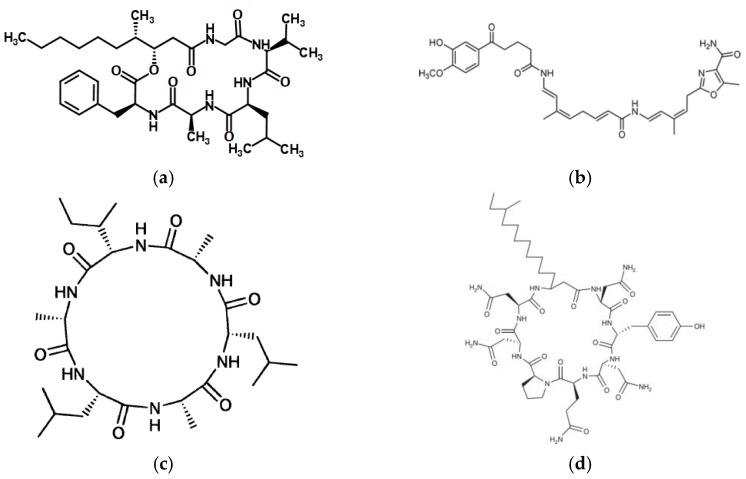

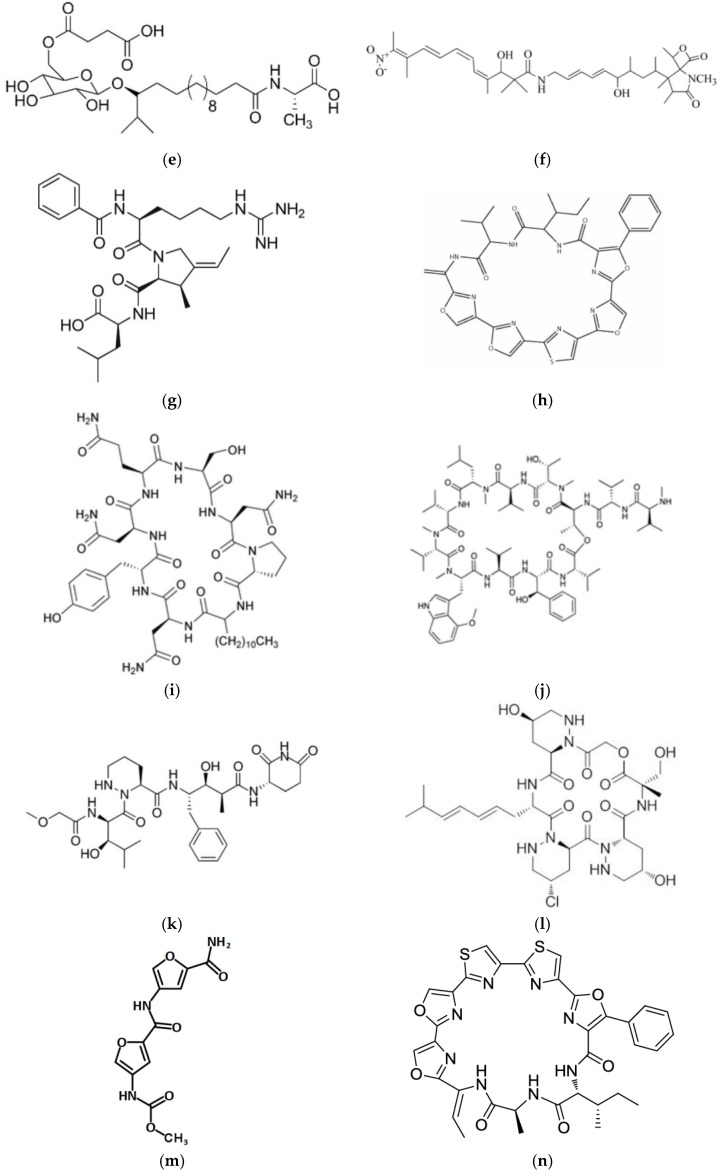
Chemical structures of anticancer nonribosomal peptides: (**a**) Arenamide A; (**b**) Ariakemicin A; (**c**) Halolitoralin A; (**d**) Mojavensin A; (**e**) Ieodoglucomide A; (**f**) Lajollamycin; (**g**) Lucentamycin A; (**h**) Mechercharmycin A; (**i**) Mixirin A; (**j**) Ohmyungsamycin A; (**k**) Padanamide B; (**l**) Piperazimycin A; (**m**) Proximicin A; (**n**) Urukthapelstatin A.

Nonribosomal peptides (NRPs) constitute secondary bioactive metabolites synthesized by an enzyme complex present only in bacteria, cyanobacteria and fungi [84]. NRPs are characterized by a lot of interesting chemical structures including d-amino acids, *N*-terminally attached fatty acid chains, *N*- and *C*-methylated residues, *N*-formylated residues, heterocyclic rings, glycosylated amino acids and phosphorylated residues [85]. Some NRPs exhibit anticancer and/or antimicrobial activity [84]. The source and biological target of anticancer nonribosomal peptides are presented in Table 4, while their chemical structures are shown in Figure 5.

### 5.1. Arenamides

Three new cyclohexadepsipeptides—named arenamides A–C—were obtained from the fermentation broth of *Salinispora arenicola* found in sea sediment (Great Astrolabe Reef, Kandavu Island chain, Fiji). Authors reported that arenamides A and B blocked TNF-induced activation with an IC_50_ value at the level of 3.7 and 1.7 μM, respectively. Moreover, inhibition of nitric oxide and prostaglandin E2 production and also moderate cytotoxic effect on human colon carcinoma (HCT-116) were detected [86].

### 5.2. Ariakemicins

The culture of the marine gliding bacterium of the *Rapidithrix* genus (Ariake Inland Sea, Japan) yielded two linear hybrid polyketide-nonribosomal peptides (ariakemicins A and B). These proteins show antimicrobial activity and contain threonine, two ω-amino-(ω-3)-methyl carboxylic acids with diene or triene units and δ-isovanilloylbutyric acid. Ariakemicins exhibit low cytotoxicity to human lung tumour cell line (A549) and baby hamster kidney cells with an IC_50_ value at the level of 42.3 and 25.4 μM, respectively [87].

### 5.3. Halolitoralins

A cyclic hexapeptide (halolitoralin A) and two cyclic tetrapeptides (halolitoralin B and C) were derived from *Halobacillus litoralis* YS3106 found in the marine sediments (Huanghai Sea, China). Halolitoralin A has a molecular mass of 575 Da and a molecular formula of C_27_H_48_O_6_N_6_, while halolitoralin B and C appealed as isomers with a chemical formula of C_23_H_42_O_4_N_4_. Presented cyclopeptides exhibit moderate activities in vitro against human gastric tumour cells (BGC) [88].

### 5.4. Heptapeptide from Paenibacillus profundus

A linear glyceryl acid derived heptapeptide (Glyceryl-d-leucyl-d-alanyl-d-leucyl-d-leucyl-l-valyl-d-leucyl-d-alanine) was produced by the culture of marine deep sediment strain SI 79 classified as *Paenibacillus profundus* sp. nov. The peptide is an antibiotic with cytotoxic activity against human melanoma cell line (SK-MEL-28) with IC_50_ = 3.07 μM after 72 h [89].

### 5.5. Ieodoglucomides

Ieodoglucomide A and B are glycolipopeptides obtained from *Bacillus licheniformis* occurring in marine sediment of Ieodo Reef (South Korea). Both peptides showed low antimicrobial activity in vitro but ieodoglucomide B demonstrated cytotoxicity against lung and stomach cancer cells (50% growth inhibition, GI_50_ = 25.18 and 17.78 μg/mL, respectively) [90].

### 5.6. Iturinic Lipopeptides

Ma et al. [91] isolated three iturinic lipopeptides from *Bacillus mojavensis* B0621A originated from pearl oyster *Pinctada martensii* in the South China Sea. Mojavensin A has a molecular formula of C_50_H_77_N_13_O_14_ and a molecular weight of 1.1 kDa. Two other isolated substances had singly and doubly-charged molecular ions. Iso-C16 fengycin B possesses a molecular weight of 1.5 kDa and 746 Da, respectively and anteiso-C17 fengycin B has 1.5 kDa and 753 Da, respectively. All three lipopeptides showed weak cytotoxic activities against human leukemia (HL-60) cell line. Mojavensin A, iso-C16 fengycin B and anteiso-C17 fengycin B inhibited the growth of HL-60 with IC_50_ of 100, 100 and 1.6 mM, respectively [91].

### 5.7. Lajollamycin

Actinomycete *Streptomyces nodosus* NPS007994 obtained from marine sediment of Scripps Canyon, La Jolla, California, USA, was reported as a source of lajollamycin. This peptide, a nitro-tetraene spiro-b-lactone-g-lactam, showed antimicrobial activity. Lajollamycin reduced in vitro the growth of the mouse melanoma cells (B16-F10) with a half maximal effective concentration (EC_50_) of 9.6 μM [92].

### 5.8. Lucentamycins

Cho et al. [93] isolated from the broth of a marine-derived actinomycete strain *Nocardiopsis lucentensis* CNR-712 3-methyl-4-ethylideneproline-containing peptides, named as lucentamycins A–D. Among them, lucentamycins A and B exhibited significant in vitro cytotoxicity against human colon carcinoma cells (HCT-116) with IC_50_ values of 0.20 and 11 μM, respectively [93].

### 5.9. Mechercharmycins

Kanoh et al. [94] obtained mechercharmycins from the *Thermoactinomyces* species YM3-251 originated from mud (Mecherchar, Republic of Palau, North Pacific Ocean). The cyclic peptide mechercharmycin A has a chemical formula of C_35_H_32_N_8_O_7_S and a molecular weight of 708 Da, whereas the linear congener mechercharmycin B has a formula of C_35_H_36_N_8_O_10_ and a molecular weight of 728 Da. Mechercharmycin A showed relatively strong antitumor activity against human lung cancer cells (A549) and human leukemia (Jurkat cells) with an IC_50_ value of 4.0 × 10^−8^ M and 4.6 × 10^−8^ M, respectively. In the case of mechercharmycin B, anticancer activity was not detected [94].

### 5.10. Mixirins

Three cyclic acylpeptides named as mixirins A-C were isolated by Zhang et al. [95] from marine bacterium *Bacillus* sp., collected from sea mud near the Arctic pole. Mixirin A has a chemical formula of C_48_H_75_N_12_O_14_, mixirin B—C_45_H_69_N_12_O_14_ and mixirin C—C_47_H_73_N_12_O_14_. The molecular weight of all compounds was about 1 kDa. Mixirins A, B and C blocked the growth of human colon tumor cell line (HCT-116) with an IC_50_ value at the level of 0.65, 1.6 and 1.26 µM, respectively [95].

### 5.11. Ohmyungsamycins

*Streptomyces* sp. isolated from a volcanic island in the Republic of Korea produced cyclic peptides ohmyungsamycin A and B. These chemical compounds contain amino acid units, such as *N*-methyl-4-methoxytrytophan, β-hydroxyphenylalanine and *N*,*N*-dimethylvaline. Both peptides showed growth inhibition against diverse cancerous cell lines obtaining an IC_50_ value in the range from 359 to 816 nM and from 12.4 to 16.8 μM, respectively. Moreover, ohmyungsamycins exhibited relatively selective anti-proliferative activity against tumor cells in comparison with normal cells [96].

### 5.12. Padanamides

In the culture of *Streptomyces* sp. isolated from the marine sediment, two highly modified linear tetrapeptides: padanamides A and B were produced. Authors demonstrated that padanamide A inhibits cysteine and methionine biosynthesis and padanamide B is cytotoxic to human leukemia (Jurkat cells) with an IC_50_ value of 30.9 μM [97].

### 5.13. Piperazimycins

Miller et al. [98] isolated three cyclic hexadepsipeptides piperazimycins A–C from the fermentation broth of *Streptomyces* sp., originated from marine sediments near the island of Guam. These substances contain rare amino acids, such as hydroxyacetic acid, α-methylserine, γ-hydroxypiperazic acid, γ-chloropiperazic acid, 2-amino-8-methyl-4,6-nonadienoic acid and 2-amino-8-methyl-4,6-decadienoic acid. All studied peptides demonstrated cytotoxicity against diverse cancer cells with a mean GI_50_ of 100 nM in the case of piperazimycin A [98].

### 5.14. Proximicins

Three novel aminofuran antibiotics (proximicins) were extracted by Fiedler et al. [99] from marine member of the rare genus *Verrucosispora*, strain MG-37. Bacterium was isolated from sediment collected in the Raune Fjord, Norway, at a depth of 250 m. Second strain of *Verrucosispora*, AB-18-032 was isolated from sediment obtained from the Sea of Japan at a depth of 289 m. Molecular formulas and weights of proximicins A, B and C are C_12_H_11_N_3_O_6_, 293 Da, C_20_H_19_N_3_O_7_, 413 Da and C_22_H_20_N_4_O_6_, 436 Da, respectively. All compounds demonstrated growth inhibition potential against cell lines of gastric adenocarcinoma (AGS, GI_50_ = 0.25–1.5 μg/mL), hepatocellular carcinoma (HepG2, GI_50_ = 0.78–9.5 μg/mL) and breast carcinoma (MCF-7, GI_50_ = 5.0–9.0 μg/mL). After 24 h of incubation of AGS cells, proximicin C caused cell arrest in the G0/G1 phase, whereas the number of apoptotic cells was increased after 40 h. Additionally, the above-mentioned substance induced upregulation of p53 and of the cyclin kinase inhibitor p21 in AGS cells [99].

### 5.15. Urukthapelstatin A

A cyclic thiopeptide-urukthapelstatin A was isolated from the cultured mycelia of *Mechercharimyces asporophorigenens* YM11-542 bacterium originated from sediments of marine lake (Urukthapel Island, Palau) [100,101]. The molecular formula of urukthapelstatin A was established as C_34_H_30_N_8_O_6_S_2_ with weight of 733 Da [101]. Urukthapelstatin A showed dose-dependent growth inhibition of human lung tumor cells (A549) with an IC_50_ value at the level of 12 nM. Presented substance seemed to be the most effective against the ovarian cancer (OVCAR-3, OVCAR-4, OVCAR-5, OVCAR-8 and SK-OV3), breast cancer (MCF-7), colon cancer (HCT-116) and lung cancer (DMS114 and NCIH460) [100].

## 6. Toxins

**Figure 6:**
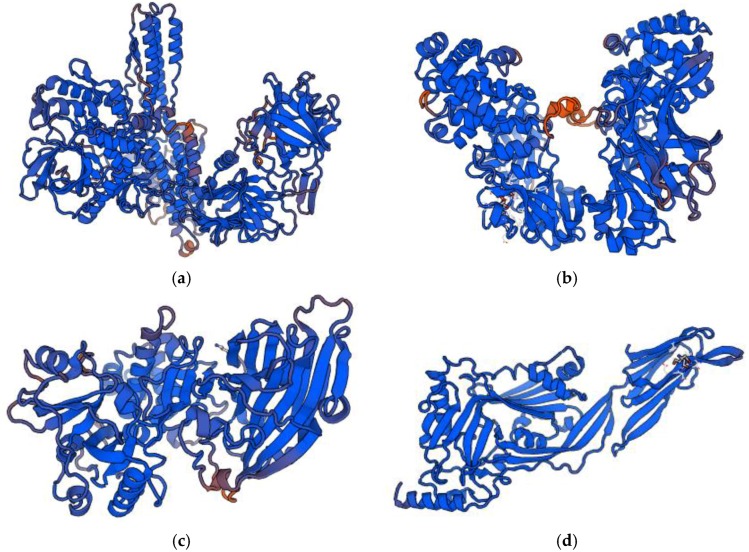
Structure models of anticancer bacterial toxins: (**a**) Botulinum neurotoxin type A; (**b**) Diphtheria toxin; (**c**) Exotoxin A; (**d**) Listeriolysin O (orig.). Sequences were downloaded from UNIPROT [62], while visualization of the toxin structures was prepared using the SWISS-MODEL modeling server [63,64].

Toxins produced by the bacteria damage host tissues directly at the site of bacterial infection or may spread throughout the body. Some toxins are tried to be used for therapeutic purposes [102]. The source and biological target of bacterial toxins with anticancer activity are presented in Table 5 and their structure models in Figure 6.

### 6.1. Botulinum Neurotoxin Type A

Botulinum neurotoxin type A, produced by strains of Clostridium botulinum, is utilized in the treatment of benign prostatic hyperplasia (BPH) due to its apoptotic activity. Toxin reduces also cell growth and proliferation of prostate cancer (PC-3 and LNCaP) cell lines [104,105]. Moreover, botulinum toxin A induces caspase-3 and -7 dependent apoptotic processes in the breast cancer cell line (T47D) [106].

### 6.2. Diphtheria Toxin

Diphtheria toxin (DT) represents an exotoxin obtained from *Corynebacterium diphtheriae*. This substance has a molecular weight of 60 kDa and its production is caused by the infection of bacteriophage B. DT is encoded by the *tox* gene of some corynebacteriophages, hence only *C. diphtheriae* isolates that contain the *tox*+ phages secrete diphtheria toxin [107]. DT exhibits the anticancer activity but with side effects, so it is utilized in the antitumor therapy in combination with other agents. The cross-reacting material 197 (CRM197) is the nontoxic mutant of diphtheria toxin that binds heparin-binding epidermal growth factor-like growth factor. It was shown that CRM197 inhibited angiogenesis and stimulated cell apoptosis of human adrenocortical carcinoma (H295R) [108]. Other substance, DTAT is DT-based immunotoxin directed to cancer vascular endothelium. DTAT exhibited in vitro strong anticancer action in the case of glioblastoma cell lines (U118MG, U373MG, U87MG) [109]. In turn, denileukin diftitox is a fusion protein designed against cells which express the IL-2 receptor. It is used as a drug named Ontak in cutaneous T cell lymphomas (CTCL) expressing CD25 [110,112].

### 6.3. Exotoxin A

Exotoxin A belongs to the main toxins produced by *Pseudomonas aeruginosa*. The molecular weight of this peptide is 66 kDa. It inhibits protein synthesis by the inactivation of elongation factor-2 (EF-2). This substance is usually utilized as an immunotoxin with different ligands [114]. Deimmunized *Pseudomonas* exotoxin cloned with human epidermal growth factor (EGF) and interleukin 4 showed activity against pancreatic cancer (PaCa-2) and selectively prevented metastasis [115]. Two exotoxin A-based immunotoxins (9.2.27PE ABT-737) caused synergistic cytotoxicity and death of melanoma cell lines (FEMX, Melmet-1, Melmet-5, Melmet-44, MelRM, MM200) associated with apoptosis [116]. In the case of exotoxin A cloned with an anti-CD133 scFv reactive (dCD133KDEL), the inhibition of cell multiplication of head and neck squamous carcinoma was observed [117].

### 6.4. Listeriolysin O

Listeriolysin O (LLO) is produced by strains of *Listeria monocytogenes*, a pathogen which develops within the cell cytosol. This chemical compound is crucial to the phagosomal escape of the bacterium into the cytoplasm [119]. The conjugated immunotoxin B3-LLO exhibited high effectiveness in removing the breast carcinoma cell lines MCF7 and SKBR-3, with an EC_50_ value at the level of 2.3 and 12.7 nM, respectively [121]. According to Stachowiak et al. [122], supernatants of *L. monocytogenes* strains showed dose-dependent cytotoxicity against the human leukemia T-lymphocyte cells (Jurkat) and human peripheral blood mononuclear cells (PBMC). Authors suggested that LLO activity is targeted more to T cells than B cells and it may give some therapeutic consequences, including T-cell lymphoma [122].

## 7. Other Proteins/Peptides

**Figure 7:**
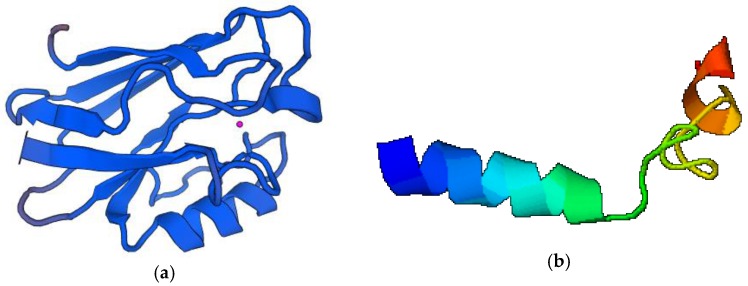
Structure model of: (**a**) Azurin; (**b**) p28 (orig.).

In this part, four bacterial proteins or peptides have been described. The source and biological activity of these anticancer substances are presented in Table 6. In turn, their structure models are shown in Figure 7.

Sequences were downloaded from UNIPROT [62]. Modeling server SWISS-MODEL [63,64] and I-TASSER [137] were used to visualization of the azurin and p28 structure, respectively.

### 7.1. Azurin

Azurin is a copper-containing protein with a molecular mass of 16 kDa, secreted by *Pseudomonas aeruginosa*. After removing the copper, the cytotoxic apo-azurin is formed [123]. Several different mechanisms of azurin anticancer activity have been proposed: (i) induction of cancer cell apoptosis or growth inhibition by forming complexes with tumor protein p53; (ii) inhibition of cancer cell growth by interfering in the receptor tyrosine kinase EphB2-mediated signaling process; (iii) inhibition of tumor growth by preventing angiogenesis through reducing VEGFR-2 tyrosine kinase activity; (iv) interferention with P-cadherin protein expression and inhibition of the growth of breast cancer cells [124]. Peptide had a strong cytotoxic effect on the breast cancer cell line (MCF-7), resulting in more than 50% increase of apoptosis and poor to other breast cancer cells (MDA-MB-157, MDD2, MDA-MB-231) [125]. In other studies, the azurin showed anticancer activity against oral squamous carcinoma cells (YD-9) [126] and melanoma cells (UISO-Mel-2) [127].

### 7.2. p28

p28 is a part of azurin (amino acids 50-77) consisting of 28 amino acids. Its molecular mass reaches 2.8 kDa [128]. p28 has an influence on the post-translational increase of p53 and p21 expression, which causes cell arrest in the G2-M phase. Also, p28 showed antiangiogenic effect and preferentially entered the human breast cancer cells (MCF-7, ZR-75-1, T47D) through a caveolin-mediated pathway [129,130]. It is interesting that this substance raised the cytotoxicity of lower doses of DNA-damaging (doxorubicin, dacarbazine, temozolomide) or antimitotic (paclitaxel, docetaxel) drugs in glioblastoma cells (U87 and LN229) and p53wt melanoma (Mel-29). The increased activity of the above-mentioned antitumor medicines in combination with p28 was facilitated through the p53/p21/CDK2 pathway [131]. p28 has recently completed two Phase I clinical trials (brain and solid tumors) [132].

### 7.3. Entap

Enterococcal anti-proliferative peptide (Entap) is produced by clinical strains of *Enterococcus* genus and has a molecular weight of 6.2 kDa. Entap demonstrated antiproliferative activity against cell lines of human gastric adenocarcinoma (AGS), colorectal adenocarcinoma (HT-29), mammary gland adenocarcinoma (MDA-MB-231), uterine cervix adenocarcinoma (HeLa) and also prostatic carcinoma (22Rv1). The activity of Entap is associated with cancer cell arrest in G1 and induction of autophagous apoptosis [114,133,134].

### 7.4. Pep27anal2

Pep27anal2, with a molecular weight of 3.3 kDa, constitutes an analogue of the signal peptide of *Streptococcus pneumoniae* (Pep27). This substance activates *S. pneumoniae* death program and exhibits antimicrobial properties [135]. Pep27anal2 gets through the cell membrane inducing caspase- and cytochrome c-independent apoptosis. Pep27anal2 inhibited proliferation of cell lines of leukemia (AML-2, HL-60, Jurkat), gastric cancer (SNU-601) and breast cancer (MCF-7) [136].

## 8. Final Remarks

In the course of evolution, host defence peptides and proteins developed in various organisms, such as bacteria [138], fungi [139], plants [140], animals [138,141] and human [142]. Some substances exhibit the multifunctional activity, for example, antimicrobial and antitumor properties [51,84,140,143,144]. However, the number of these bioactive compounds known so far is a relatively small. Investigations concerning their isolation, cognition and application are just the tip of the iceberg. In the present review, we described 37 bacterial chemical compounds or groups of compounds with anticancer activity. On the other hand, there are about 30,000 known, cultured bacterial species [145] and about 109,000 of operative taxonomic units of bacteria are found on the basis of the 16S rRNA study [146]. It can be supposed that some of them constitute a potential source of new biologically active agents.

Nowadays, marine organisms are increasingly important in terms of isolated chemical compounds with antibacterial, antiviral and anticancer activity [147]. A large group of anticancer peptides was obtained from bacteria occurring in marine sediments [86,87,88,89,90,91,92,93,94,95,96,97,98,99,100,101]. In this work, we presented 15 proteins or groups of them secreted by marine bacteria. However, only the first research conducted by the authors who have isolated these substances are usually available. In most cases, there are no continuations of these studies or other investigations.

Among bacteriocins showing the antitumor action, we reported 8 peptides and proteins (individual compounds or groups of compounds). These substances belong to the better examined constituents. Some of them are produced by fermentation [148] of lactic acid bacteria (LAB) belonging to *Lactobacillus*, *Lactococcus*, *Bifidobacterium*, *Leuconostoc*, *Streptococcus* or *Pediococcus* genera [46]. Nisin is the best known bacteriocin, which in 1969 was approved by the FAO/WHO as a safe food additive. At the present, the above-mentioned bacteriocin is used as a natural preservative in over 50 countries [149]. The literature data show that milk products containing probiotic strains, for example kefir, can inhibit proliferation of cancer cells and induce apoptosis [150].

The combination of Omics techniques with virtual screening and computational methods may contribute to the development of the discussed research direction. These methods can be utilized for modification of the already known anticancer proteins or the selection of new chemical compounds with antitumor activity [151]. The other option is using of the peptide-based drug conjugates that affect the reduction of side effects in cancer patients [152]. It should be added that four described in this review antibiotics (actinomycin D, bleomycin, doxorubicin, mitomycin C) and diphtheria toxin are already utilized as medicines [30,32,33,39,112,114] and p28 has recently completed two Phase I clinical trials [132]. The others have to wait for further investigations.

To sum up, bacteria constitute a valuable and, at the same time, very poorly known source of biologically active substances, including anticancer proteins and peptides. Studies of the majority of bacterial anticancer proteins/peptides end in the in vitro stage and only single ones undergo the entire procedure, from in vitro by clinical trial to registration and use as medicines.

## Figures and Tables

**Table 1:** The origin and biological activity of anticancer antibiotics.

No.	Protein/Peptide	Source	Biological Target: Human Cancer Cells	IC_50_	References
1	Actinomycin D	*Actinomyces antibioticus*	Wilms cancer, Ewing sarcoma, neuroblastomas, trophoblastic tumours	from 0.4 nM to 0.42 µM	[12,13,14,15]
2	Bleomycin	*Streptomyces verticillus*	head and neck squamous cell carcinomas, Hodgkin’s disease, non-Hodgkin’s lymphoma, testicular carcinomas, ovarian cancer, malignant pleural effusion	from 25.2 nM to 2.93 mM	[12,16,17,18,19]
3	Doxorubicin	*Streptomyces peucetius* var. *caesius*	acute lymphoblastic leukaemia, acute myeloblastic leukaemia, Wilms’ tumour, neuroblastoma, soft tissue and bone sarcomas, breast carcinoma, ovarian carcinoma, transitional cell bladder carcinoma, thyroid carcinoma, gastric carcinoma, Hodgkin’s disease, malignant lymphoma, bronchogenic carcinoma, oral squamous carcinoma	from 548.2 nM to 44.7 µM	[12,20,21,22,23]
4	Mitomycin C	*Streptomyces caespitosus*	cancers of the head and neck, lungs, breast, cervix, bladder, colorectal and anal carcinomas, hepatic cell carcinoma, melanoma, stomach and pancreatic carcinomas	from 9.48 nM to 249 µM	[12,24,25,26]

IC_50_—half maximal inhibitory concentration.

**Table 2:** The origin and biological activity of anticancer bacteriocins.

No.	Protein/Peptide	Source	Biological Target: Human Cancer Cell Lines	IC_50_	References
1	Bovicin HC5	*Streptococcus bovis* HC5	breast adenocarcinoma (MCF-7), liver hepatocellular carcinoma (HepG2)	279.4–289.3 µM	[41,47]
2	Colicins A and E1	*Escherichia coli*	breast carcinoma (MCF7, ZR75, BT549, BT474, MDA-MB-231, SKBR3, T47D), osteosarcoma (HOS), leiomyosarcoma (SKUT-1), fibrosarcoma (HS913T)	n.d.	[41,48]
3	Laterosporulin 10	*Brevibacillus* sp. strain SKDU10	cervical cancer (HeLa), embryonic kidney cancer (HEK293T), fibrosarcoma (HT1080), lung carcinoma (H1299) breast cancer (MCF-7)	n.d.	[49]
4	Microcin E492	*Klebsiella pneumoniae* RYC492	cervical adenocarcinoma (HeLa), acute T cell leukaemia (Jurkat), Burkitt’s lymphoma (Ramos), B-lymphoblastoid cells (RJ2.25)	n.d.	[50,51]
5	Nisin A	*Lactococcus lactis*	head and neck squamous cell carcinoma (UM-SCC-17B, UM-SCC-14A, HSC-3), breast adenocarcinoma (MCF-7), liver hepatocellular carcinoma (HepG2), acute T cell leukaemia (Jurkat)	105.5–225 µM	[40,47,52,53]
6	Nisin ZP	*Lactococcus lactis*	head and neck squamous cell carcinoma (UM-SCC-17B, HSC-3)	n.d.	[54]
7	Pediocin CP2	*Pediococcus acidilactici* MTCC 5101	mammary gland adenocarcinoma (MCF-7), hepatocarcinoma (Hep G2), cervical adenocarcinoma (HeLa)	n.d.	[55,56]
8	Pediocin K2a2-3	*Pediococcus acidilactici* K2a2-3	colon adenocarcinoma (HT29)	n.d.	[57]
9	Plantaricin A	*Lactobacillus plantarum* C11	T cell leukaemia (Jurkat)	n.d.	[58]
10	Pyocin S2	*Pseudomonas aeruginosa* 42A	hepatocellular carcinoma (HepG2), multiple myeloma (Im9), cervical adenocarcinoma (HeLa), embryonal ovary carcinoma (AS-II)	n.d.	[59,60]

IC_50_—half maximal inhibitory concentration, n.d.—no data.

**Table 3:** The origin and biological activity of anticancer bacterial enzymes.

No.	Protein/Peptide	Source	Biological Target: Human Cancer Cells/Cell Lines	IC_50_	References
1	Arginine deiminase	*Mycoplasma hominis*, *M. arginini*	hepatocellular carcinoma (HCC), prostate cancer (CWR22Rv1), glioblastoma (HROG02, HROG05, HROG10, HROG17)	1.95 µg/mL	[67,68,69,70,71]
2	l-asparaginase	*Escherichia coli*, *Erwinia* sp.	paediatric medulloblastoma (DAOY), glioblastomas (GBM-ES, U87), acute lymphoblastic leukaemia (ALL, HL60, MOLT-3, MOLT-4), myeloblastic leukaemia, acute T cell leukaemia (Jurkat), Hodgkin and non-Hodgkin lymphomas, myelosarcoma, multiple myeloma, extranodal NK/T cell lymphoma, ovarian carcinomas	0.39–90 µg/mL	[72,73,74,75,76,77,78,79,80]

IC_50_—half maximal inhibitory concentration.

**Table 4:** The origin and biological activity of anticancer nonribosomal peptides.

No.	Protein/Peptide	Source	Biological Target: Cancer Cells	IC_50_	References
1	Arenamides A, B	*Salinispora arenicola*	human colon carcinoma (HCT-116)	1.7–3.7 µM	[86]
2	Ariakemicins A, B	*Rapidithrix* sp.	human lung cancer (A549)	25.4–42.3 µM	[87]
3	Halolitoralins A–C	*Halobacillus litoralis* YS3106	human gastric tumour (BGC)	n.d.	[88]
4	Heptapeptide	*Paenibacillus profundus*	human melanoma (SK-MEL-28)	3.07 µM	[89]
5	Ieodoglucomide B	*Bacillus licheniformis*	human lung cancer, human stomach cancer	n.d.	[90]
6	Iso-C16 fengycin B, anteiso-C17 fengycin B, mojavensin A	*Bacillus mojavensis* B0621A	human leukaemia (HL-60)	1.6–100 mM	[91]
7	Lajollamycin	*Streptomyces nodosus* NPS007994	mouse melanoma (B16-F10)	n.d.	[92]
8	Lucentamycins A, B	*Nocardiopsis lucentensis* CNR-712	human colon carcinoma cells (HCT-116)	0.2–11 µM	[93]
9	Mechercharmycin A	*Thermoactinomyces* sp. YM3-251	human lung cancer cells (A549), human leukaemia (Jurkat)	400–460 µM	[94]
10	Mixirins A–C	*Bacillus* sp.	human colon tumour (HCT-116)	0.65–1.6 µM	[95]
11	Ohmyungsamycins A and B	*Streptomyces* sp.	diverse cancer cells	from 359 nM to 16.8 µM	[96]
12	Padanamide A, B	*Streptomyces* sp.	human leukaemia (Jurkat)	30.9–90.7 µM	[97]
13	Piperazimycins A–C	*Streptomyces* sp.	multiple tumour cell lines	n.d.	[98]
14	Proximicins A–C	*Verrucosispora* sp. MG-37 and AB-18-032	human gastric adenocarcinoma (AGS), human hepatocellular carcinoma (HepG2), human breast carcinoma (MCF 7)	n.d.	[99]
15	Urukthapelstatin A	*Mechercharimyces asporophorigenens* YM11-542	human lung cancers (A549, DMS114, NCIH460), ovarian cancers (OVCAR-3, OVCAR-4, OVCAR-5, OVCAR-8, SK-OV3), breast cancer (MCF-7), colon cancer (HCT-116)	12 nM	[100,101]

IC_50_—half maximal inhibitory concentration, n.d.— no data.

**Table 5:** The origin and biological activity of anticancer bacterial toxins.

No.	Protein/Peptide	Source	Biological Target: Human Cancer Cell Lines	IC_50_	References
1	Botulinum neurotoxin type A	*Clostridium botulinum*	prostate cancer (PC-3, LNCaP), breast cancer (T47D), neuroblastoma (SH-SY5Y)	0.54–300 nM	[103,104,105,106]
2	Diphtheria toxin	*Corynebacterium diphtheriae*	adrenocortical carcinoma (H295R), glioblastomas (U118MG, U373MG, U87MG), cutaneous T cell lymphomas (CTCL), breast carcinoma (MCF 7), cervical adenocarcinoma (HeLa)	0.55–2.08 µg/mL	[107,108,109,110,111,112]
3	Exotoxin A	*Pseudomonas aeruginosa*	pancreatic cancer (PaCa-2), melanomas (FEMX, Melmet-1, Melmet-5, Melmet-44, MelRM, MM200), head and neck squamous carcinomas, Burkitt’s lymphoma (Daudi, CA46), leukemias (EHEB, MEC1)	0.3–8.6 ng/mL	[113,114,115,116,117]
4	Listeriolysin O	*Listeria monocytogenes*	breast carcinomas (MCF7, SKBR-3), leukemia T-lymphocytes (Jurkat)	from 50 pM to 0.1 nM, in conjugates	[118,119,120,121,122]

IC_50_—half maximal inhibitory concentration.

**Table 6:** The origin and biological activity of other anticancer bacterial proteins/peptides.

No.	Protein/Peptide	Source	Biological Target: Human Cancer Cells/Cell Lines	IC_50_	References
1	Azurin	*Pseudomonas aeruginosa*	breast cancer (MCF-7, MDA-MB-157), oral squamous carcinoma (YD-9), melanoma (UISO-Mel-2)	32–53 µM	[123,124,125,126,127]
2	p28	*Pseudomonas aeruginosa*	breast cancer (MCF-7, ZR-75-1, T47D), glioblastoma (U87, LN229), melanoma (Mel-29), brain tumors	n.d.	[128,129,130,131,132]
3	Entap	*Enterococcus* sp.	gastric adenocarcinoma (AGS), uterine cervix adenocarcinoma (HeLa), mammary gland adenocarcinoma (MDA-MB-231), prostate carcinoma (22Rv1), colorectal adenocarcinoma (HT-29)	n.d.	[114,133,134]
4	Pep27anal2	*Streptococcus pneumoniae*	leukemia (AML-2, HL-60, Jurkat), gastric cancer (SNU-601), breast cancer (MCF-7)	10–29 µM	[135,136]

IC_50_—half maximal inhibitory concentration, n.d.—no data.
